# A Conservative Level Set Approach to Non-Spherical Drop Impact in Three Dimensions

**DOI:** 10.3390/mi13111850

**Published:** 2022-10-28

**Authors:** Xu Pan, Ying Wang, Mingguang Shen

**Affiliations:** 1 Key Laboratory of Concrete and Prestressed Concrete Structures of Ministry of Education, School of Civil Engineering, Southeast University, Nanjing 211189, China; 2 School of Mathematics and Statistics, Yancheng Teachers University, Yancheng 224002, China

**Keywords:** conservative level set method, non-spherical drop impact, splashing

## Abstract

A recently developed conservative level set model, coupled with the Navier-Stokes equations, was invoked to simulate non-spherical droplet impact in three dimensions. The advection term in the conservative level set model was tackled using the traditional central difference scheme on a half-staggered grid. The pressure velocity coupling was decoupled using the projection method. The inhouse code was written in Fortran and was run with the aid of the shared memory parallelism, OpenMP. Before conducting extensive simulations, the model was tested on meshes of varied resolutions and validated against experimental works, with satisfyingly qualitative and quantitative agreement obtained. The model was then employed to predict the impact and splashing dynamics of non-spherical droplets, with the focus on the effect of the aspect ratio. An empirical correlation of the maximum spread factor was proposed. Besides, the number of satellite droplets when splashing occurs was in reasonable agreement with a theoretical model.

## 1. Introduction

Drop impact is ubiquitous in the world, from water droplets dripping from a faucet to the molten particles colliding at a high speed with a cold substrate in plasma spraying. The outcome of drop impact depends on a number of factors, such as the impacting speed, contact angle, drop diameter, and the like. Generally, when the impacting speed is fairly high, splashing would take place. It occurs in a wide range of industrial applications and of natural phenomena, such as plasma spraying, additive manufacturing, and raindrops impacting the earth [1,2,3]. Splashing could be divided into two categories: Prompt splashing and corona splashing. The former takes place in the early stages of spreading, mostly on a solid surface, while the latter features a high rising angle that is made between the spreading front and a substrate [4]. Splashing on one hand is to be avoided, as in the 3D printing, whereas on the other, it is desired as in pesticide spraying, where it could render more crop leaves sprayed.

Numerical simulations of drop impact could be done using two mainstream methods: Moving grid method and fixed grid method [5]. The former is with high accuracy but is complained about the complex algorithm involved. Drop impact is a typical two-phase flow where the moving interface is to be tracked. As a moving grid method, the front tracking method boasts of a fixed, but not always, number of nodes deployed along an interface so that boundary conditions across it could be precisely defined. However, this technique requires remeshing the grid and has difficulties in dealing with topological changes [6]. The fixed grid method often employs a stationary Cartesian grid, coupled with a phase indicator equation, to track an interface. The phase indicator takes on different forms, such as the volume of fluid, the phase field, and the level set [7].

As one of the fixed grid methods, the level set method tracks the interface between two different phases using the signed distance function, which is exactly zero at the interface. It was first proposed in [8] as a purely mathematical model to trace the interface between two phases. The level set method defines a sharp interface, but the signed distance function varies smoothly across the interface so that the computation of curvature is not a matter of concern. However, the incorporation and computation of surface tension into the momentum equation poses a major challenge for the volume of fluid method, since the phase indicator is discontinuous across the interface.

On the other hand, for two-phase flow simulations, mass conservation is required for each component. Due to its nature and the numerical scheme used to discretize the advection equation, the volume of fluid method strictly ensures mass conservation. However simple the level set method is, mass is notoriously not conserved so that re-initialization is entailed to ensure the property of the signed distance function. With concerted efforts, conservative level set models were proposed, improving greatly in conserving mass. In the conservative model by [9], the signed distance function was replaced with a hyperbolic function, with an extra re-initialization equation solved to ensure a stable solution. While in the work of [10], only a single advection equation is to be solved and it could be written in a conservative form, thus greatly reducing computation time and simplifying the algorithm. As the hyperbolic function is found in the steady state solutions to the phase field method, the conservative level set model could be seen as a diffuse interface model, whose interface has a thin but finite thickness. The conservative level set model is only second order, which is more amenable to explicit finite difference solutions than its counterpart, the fourth order Cahn-Hilliard phase field model.

Since the development of the conservative level set model, its application in a number of areas has been reported. Jain et al. [11] proposed a novel numerical model for two-phase immiscible compressible flows. Howard et al. [12] developed a model for *N*-phase flow with a free-energy-based surface tension. Mirjalili et al. [13] invented a model to account for both heat and mass transfer in two-phase flows. Huang and Zhang [14] studied drop wetting on a curved surface in two dimensions using another kind of conservative level set method. Recently, the application of the conservative level set method to drop impact with splashing could be found in [15,16], but few have been devoted to non-spherical drop impact, to the best knowledge of the authors. The study on non-spherical drop impact was mainly reported in [17,18] by the same group, but their numerical study was focused on the control of drop bouncing using a non-spherical drop. They utilized the volume of fluid method to capture the liquid-gas interface and the impacting regime, if denoted using the *We = ρV^2^D/σ_s_* number (*ρ* is density, *V* is impacting velocity, *D* is drop diameter, and *σ_s_* is surface tension coefficient) that is a measure of relative importance between inertia and surface tension, falls on the order of *O*(10). For regimes of *We*~100 of non-spherical drop impact onto a dry surface, few studies have been reported. Nevertheless, this regime is crucial, since with increased *We* number interesting phenomenon like drop splashing may occur. Therefore, this paper is devoted to the simulation of non-spherical drop impact dynamics using a conservative level set model, with an emphasis on drop splashing. Another novelty of the paper lies in the discretization of the convection terms in the transport equation, where a specific central difference scheme is employed [19]. This partially inspired the authors to develop an inhouse code to test the specific scheme. By comparing numerical outcomes with experimental works, we find a satisfying agreement. Besides, a prolate drop may inhibit the prompt splashing in the early spreading stage.

## 2. Mathematical Statement

**Figure 1:**
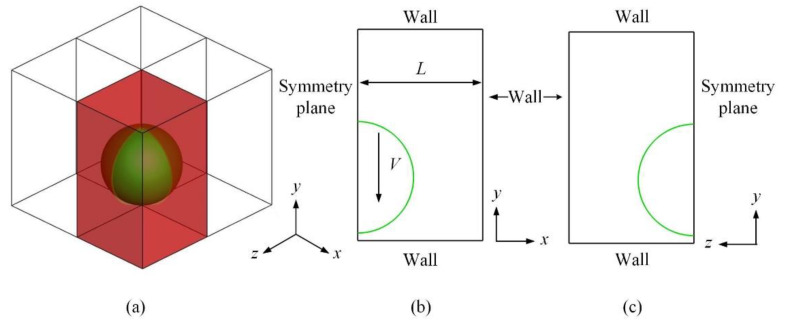
Schematic of the problem with boundary conditions defined. (**a**) shows the computational domain in three dimensions; (**b**) gives the cross-sectional view in the *xy* plane; (**c**) provides the cross-sectional view in the *zy* plane.

Let us consider a liquid drop impacting onto a solid surface at a certain velocity, as shown schematically in Figure 1. Initially, the drop is spherical, but would deviate from spherical while approaching the substrate surface. This is due to the building up of gas pressure beneath the drop. Once touching upon the substrate, radially outward motion, or the spreading process, would be promoted by the release of pressure gradient. Meantime, various elements would influence the spreading process, such as contact angle, surrounding gas viscosity, and drop inertia. The involved interfacial movement, however complex, could be taken care of in the following governing equations.

### 2.1. Level Set Equation

To track the liquid-gas interface, a one-step level set model proposed in [10] is adopted, combining the advection and the re-initialization steps into one and taking the form of

(1) $$\phi_{t}+u\cdot \nabla \phi =\nabla \cdot \left[\omega \left(\epsilon \nabla \phi -\phi \left(1-\phi \right)\frac{\nabla \phi }{\left|\nabla \phi \right|}\right)\right]$$

in which ϕ is the level set with ϕ=1 defining a gas and ϕ=0 a liquid, with the value in between indicating the diffuse interface. **u** is velocity and ∇=∂()/∂xi+∂()/∂yj+∂()/∂zk. The subscript *t* means partial derivative with respect to time, and |∇ϕ| is the norm of the gradient of the level set. The first term on the right hand side (RHS) is the artificial diffusion and the second the artificial compression. In other words, the first is to diffuse the interface while the second to sharpen it. ω gives the strength of re-initialization and ϵ measures interface thickness. In the absence of fluid motion and in the steady state, the solution to Equation (1) in one dimension takes the following form

(2) $$\phi \left(x\right)=\frac{1}{2}\left[1+\tanh\left(\frac{x}{2\epsilon }\right)\right]$$

where *x* is the coordinate perpendicular to the liquid-gas interface. If the diffuse interface is defined in the range of 0.05 < *ϕ* < 0.95, then it spans around 6ϵ.

### 2.2. Flow Field Equation

The flow field is composed of two distinct fluids, each of which is governed by the mass and the momentum conservation equations. Besides, the boundary conditions across the interface should also be enforced. However, under the framework of the level set method, a single set of equations applicable to the whole flow field could be realizable by expressing the field parameters as functions of the level set ϕ. Besides, it is assumed that the fluids are incompressible and Newtonian in this paper. Therefore, the governing equations for this complex liquid-gas flow show up as

(3) ∇·u=0

(4) $$\rho \left(\phi \right)\left(u_{t}+u\cdot \nabla u\right)=-\nabla p+\nabla \cdot \sigma +\rho \left(\phi \right)g+F_{lsm}$$

where p is pressure, $\sigma =\mu \left(\phi \right)\left[\nabla u+{\left(\nabla u\right)}^{T}\right]$ is the Newtonian stress tensor, μ is viscosity, and g is the local gravitational acceleration. Surface tension force is converted into an equivalent volumetric force in the fourth term on the RHS of Equation (4). Note that in Equation (5) $\sigma_{s}$ stands for the surface tension coefficient.

(5) $$F_{lsm}=\sigma_{s}\nabla \cdot \left(-\frac{\nabla \phi }{\left|\nabla \phi \right|}\right)\nabla \phi$$

The implicit tracking of interfaces inherent in the level set method allows for smooth transition of field variables, hence thermophysical properties could be recast as functions of the level set ϕ (e.g., $\rho \left(\phi \right)=\left(\rho_{g}-\rho_{l}\right)\phi +\rho_{l}$ where g denotes gas and l liquid).

### 2.3. Boundary Conditions

Thanks to the azimuthal symmetry of the impacting process, only a quarter of the computational domain needs calculating, as shown in Figure 1. Other boundaries are set to be walls, where pressure has a vanished gradient along the unit outward normal, as the chemical potential does. For the phase indicator ϕ, all the borders embrace homogeneous Neumann conditions, except for the bottom where the contact angle is prescribed. α is the static contact angle formed around the contact line [20,21], and the subscripts *x* and *y* stand for partial derivatives with respect to them, respectively.

(6) $$\tan\left(\frac{\pi }{2}-\alpha \right)=\frac{\phi_{y}}{\sqrt{\phi_{x}^{2}+\phi_{z}^{2}}}$$

### 2.4. Numerical Procedures

**Figure 2:**
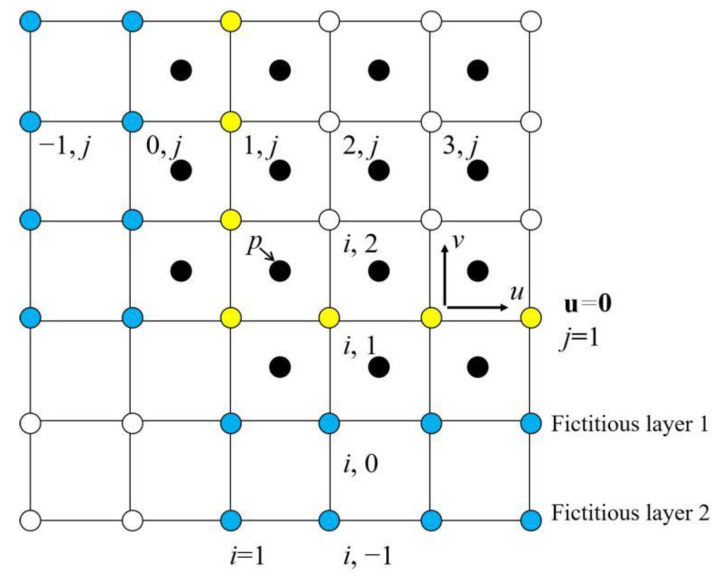
A half-staggered grid (only a corner shown herein) with two layers of ghost points. The circle in yellow denotes the boundary where the no slip condition is imposed, the circle in blue signals the ghost points and the circle in black stores the value of pressure. The points are indexed from *i* = 1 in the *x* direction and *j* = 1 in the *y* direction.

The above equations, Equations (1), (3) and (4), are discretized using a finite difference method on a half-staggered grid, with pressure stored at the cell center and others at the cell vertices, as shown in Figure 2 where for the sake of convenience and simplicity a two-dimensional version is shown. It is noticed that the convection and the diffusion terms in Equation (1) are approximated according to [19] as shown in Equations (7) and (8), instead of the upwinding scheme.

(7) $$u\cdot \nabla \phi ≅u_{i,j,k}\frac{\phi_{i+1,j,k}-\phi_{i-1,j,k}}{2\Delta x}+v_{i,j,k}\frac{\phi_{i,j+1,k}-\phi_{i,j-1,k}}{2\Delta y}+w_{i,j,k}\frac{\phi_{i,j,k+1}-\phi_{i,j,k-1}}{2\Delta z}$$

(8) $$\nabla \cdot \left[\phi \left(\phi -1\right)n\right]≅\frac{\phi_{i+1,j,k}\left(\phi_{i+1,j,k}-1\right)n_{i+1,j,k}-\phi_{i-1,j,k}\left(\phi_{i-1,j,k}-1\right)n_{i-1,j,k}}{2\Delta x}$$

where $n=\phi_{x}/\left|\phi_{x}\right|i$ is the unit normal if only the *x* direction is under consideration, and **i** is the unit normal in the *x* direction. And $n_{i+1,j,k}$ is discretized as follows, and the same could be applied to $n_{i-1,j,k}$.

(9) $$n_{i+1,j,k}=\frac{\phi_{i+2,j,k}-\phi_{i,j,k}}{2\Delta x}/\left|\frac{\phi_{i+2,j,k}-\phi_{i,j,k}}{2\Delta x}\right|$$

When dealing with three dimensions, the norm in the denominator in Equation (9) should be augmented with the counterpart in the *y* and *z* directions. In calculating the divergence in Equation (8) for the border points of *i* = 1, the value of *n_0,j,k_* is to be used. Further, when updating the value of *n_0,j,k,_* one needs to know the value on *i* = −1. Hence there is a need to arrange two layers of ghost points to prescribe the boundary conditions, as shown in Figure 2. In the *x* direction, suppose the border points are denoted as $\phi_{nx,j,k}$ and $\phi_{1,j,k}$, where *nx* is the number of total grid points in the *x* direction and is also the index of the last point. If imposing a no flux condition therein, one has

(10) $$\phi_{nx+2,j,k}=\phi_{nx-2,j,k},\;\phi_{nx+1,j,k}=\phi_{nx-1,j,k}$$

(11) $$\phi_{-1,j,k}=\phi_{3,j,k},\;\phi_{0,j,k}=\phi_{2,j,k}$$

The same way could be employed in the *z* and *y* directions. However, on the substrate surface (*j* = 1) where the contact angle is specified, $\phi_{i,0,k}$ is prescribed via Equation (6). Besides, $\phi_{i,-1,k}$ is extrapolated according to

(12) $$\phi_{y}≅\frac{\phi_{i,2,k}-\phi_{i,0,k}}{2\Delta y}≅\frac{3\phi_{i,1,k}-4\phi_{i,0,k}+\phi_{i,-1,k}}{2\Delta y}$$

It is to be noticed that the discretization schemes involved in the two identities in Equation (12) are of the same order, and that the first is the normal second order central difference scheme. The second is a one-sided difference scheme, using the value on and below *j* = 1. And only when $\phi_{i,0,k}$ is updated from the first identity, could the value of $\phi_{i,-1,k}$ be renewed through the second.

**Figure 3:**
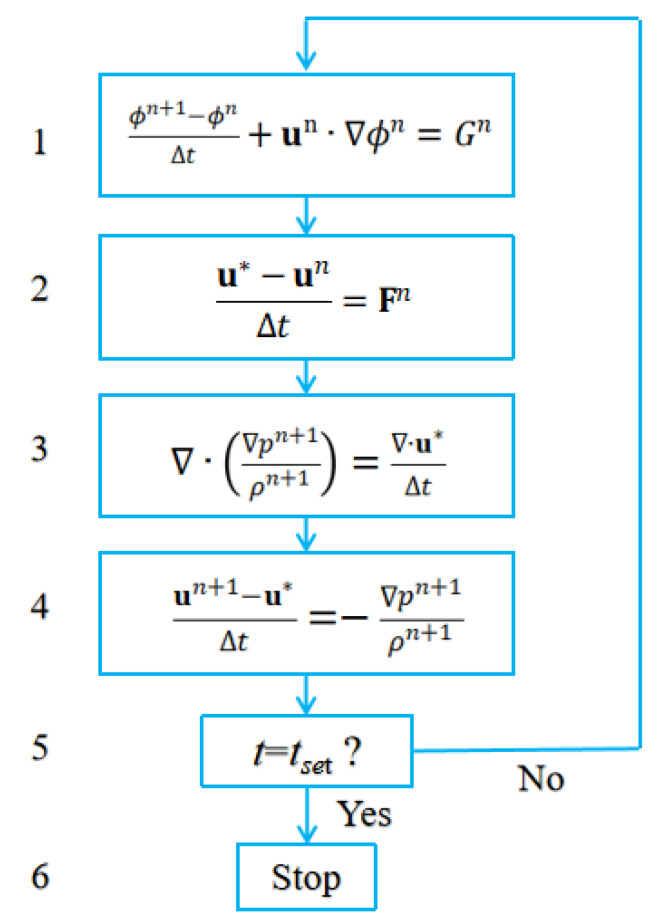
Flow chart of the current algorithm. The superscript *n* denotes the previous time level, while *n* + 1 denotes the current time level. *t_set_* represents the time duration for computation. *G^n^* stands for the term on the RHS of Equation (1). *F^n^* includes all terms in the momentum equation except for the pressure gradient term. u* is the interim velocity.

In addition, the forward Euler scheme is employed for transient terms and second order central difference scheme for diffusion terms in the Navier-Stokes equations. Convection terms are approximated using the upwinding scheme. A first order projection method is utilized to solve the pressure-velocity coupling. The flow chart of the current algorithm is given in Figure 3. To ensure the boundedness of the level set, which stipulates that the level set should be strictly between 0 and 1, any point value larger than unity or smaller than zero is forced to be unity or zero in step 1, respectively.

**Figure 4:**
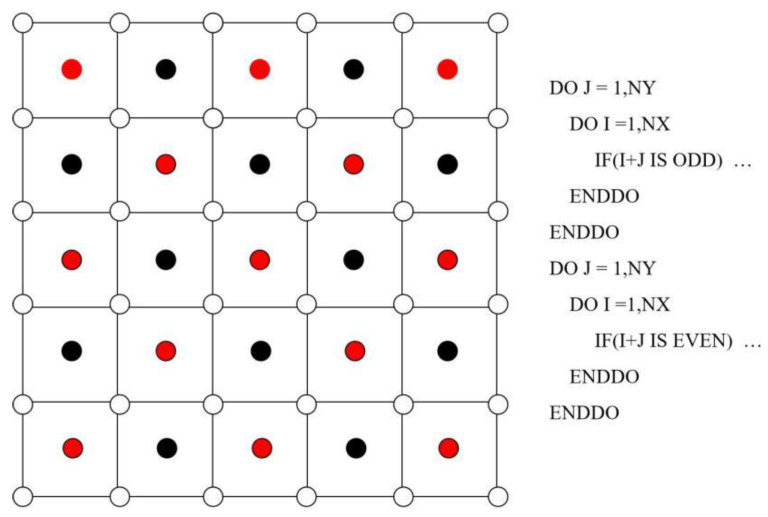
The Red/Black ordering and the pseudo code for its implementation.

As shown in Figure 3, the solution step starts from the updating of the level set, and then proceeds to the flow field. In the resulted pressure Poisson equation in step 3, a parallel version of the SOR algorithm based on the Red/Black ordering was employed, as shown in Figure 4. The SOR iteration is an extension of the Gauss-Seidel iteration. If *p* is moved from *p^i^* to *p^i+^*^1^ in one iteration, and the difference *p^i+^*^1^ − *p^i^* is supposed to take us closer to the real value of *p*, then it would make sense to go farther in this direction by update *p* according to *p^i+^*^1^ = *p^i^ + λ*(*p^i+^*^1^ − *p^i^*), where *λ* is the relaxation factor and is set to be around 1.5 throughout the paper. Besides, the code is written in Fortran and accelerated with the shared memory parallelism, OpenMP. The time step is chosen according to the CFL condition to ensure numerical stability for an explicit finite difference method. The time step is on the order of magnitude of 10^−6^ s throughout the paper.

Figure 4 shows the Red/Black ordering in two dimensions. The grid points storing pressure are divided into two groups such that only the neighboring red points are used when updating the black points. Once the black points are renewed, the red points are going to be updated using the newest pressure values stored only at the black points, and vice versa.

## 3. Results and Discussion

As the model, including its discretization and solving method, has been elaborated on, one is in a position to test and utilize the model for future predictions. Since the paper is dealing with contact line dynamics, one major issue is testing the numerical configuration of contact angle, which is put in Section 3.1. After that, a mesh independence study is followed to choose an appropriate mesh size to be used in the following sections. Moreover, it is necessary to compare the numerical outcome with the experiment to validate the model. Then, extensive simulations regarding non-spherical drop impact could be run. Accordingly, the overall framework is first to validate the model and then to employ the model.

The thermophysical quantities used in this paper are listed in Table 1. It is noted that the re-initialization strength *ω* is fixed to the initial impacting velocity *V* so that it varies from section to section.

### 3.1. Contact Angle Validation

Contact angle matters in simulations dealing with contact line motion. In this paper, it is specified in a geometric way and is forced to keep a specified value *α* as in Equation (6) all the way through drop spreading and retracting. Nevertheless, the contact angle is changing as a drop spreads or retracts, hence the notion of a dynamic contact angle. The setting up of a dynamic contact angle is usually related to the static contact angle *α* and the contact line velocity, which is often defined as the time derivative of the wetted region [22]. As the specific function relating the dynamic contact angle with other variables, like the contact line velocity, varies in the literature [23], the authors seek not to employ a dynamic contact angle herein, but stick to a static contact angle.

**Figure 5:**
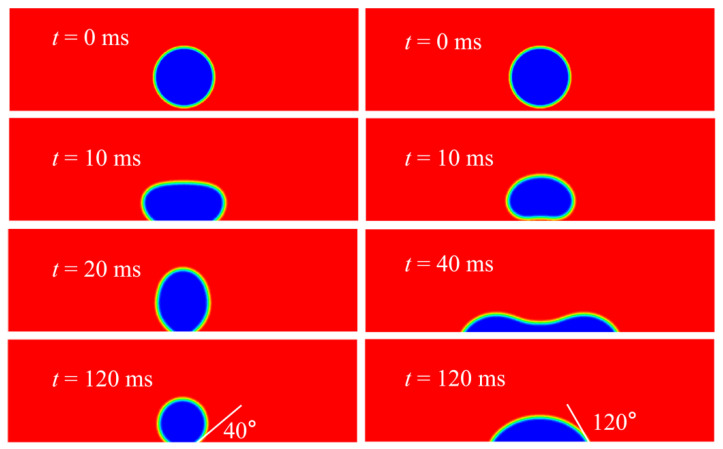
Contact angle validation on surfaces of varied wettabilities.

Before moving on to massive numerical simulations, the contact angle formulation in Equation (6) was validated. The simulations were run in two dimensions, where a Tin droplet of diameter 2.7 mm impacts onto a solid surface, whose surface wettability varies from hydrophilic to hydrophobic. The impinging velocity is 0.1 m/s and the contact angle set to 60° on a hydrophilic surface and 140° on a hydrophobic one. The number of grid points totals to 341 × 101, corresponding to a physical size of 17 mm × 5 mm. The spatial step Δ*x =* Δ*y* = 0.05 mm. Besides, there are 27 cells across the drop radius. The numerical outcome is presented in Figure 5.

Figure 5 shows that in the steady state, the prescribed contact angle is measured to be in satisfying agreement with what has been set up numerically before. As the static contact angle varies, the final drop shape differs greatly, and the maximum spread is larger in the wetted surface than in the partially wetted one.

### 3.2. Mesh Sensitivity Study

**Figure 6:**
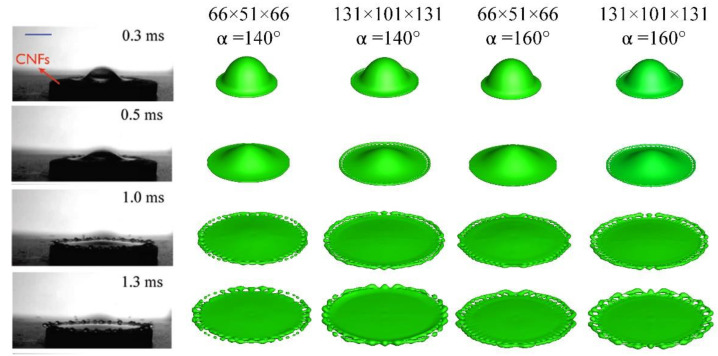
Mesh sensitivity study.

This section helps determine the mesh resolution needed to capture the phenomena in length scales of interest. As one of the focuses of the paper is on splashing dynamics, the daughter droplet ejected during splashing should be well captured. To that end, a water droplet of diameter 1.86 mm is made impinge at 2.98 m/s onto a solid substrate. The simulation was done on two meshes of different resolutions, with the effect of contact angle taken into account as well. The computational domain is *LX* × *LY* × *LZ* = 3.25 mm × 2.5 mm × 3.25 mm, with the length in the direction of gravitation being 2.5 mm. The computational domain has been examined to preclude any side effect. Besides, the droplet is released 0.4 mm above the substrate to allow for the building up of stagnation pressure. The numerical outcome, shown in Figure 6, is also compared with the experiment, which could be found in [24].

In Figure 6, the first column represents the experimental work, the second and fourth display the numerical outcome in the mesh of ∆*x* = 5 × 10^−5^ m, and the third and fifth give the numerical prediction in the mesh of ∆*x* = 2.5 × 10^−5^ m. It is to be noted that the spatial increments in the three directions are equal, and that the number of grid points, for instance, 66 × 51 × 66, corresponds to only a quarter of the computational domain. Also, thanks to the symmetric spreading process, only that portion of domain needs computing.

At *t* = 0.3 ms, the droplet just touches the substrate, with a thin film formed around the periphery. The thin film is linked to the kink produced before the droplet contacts the substrate. Initially, the air beneath the droplet tries to dimple the bottom of the droplet while escaping radially outwards. However, a tiny portion of air is bound to be trapped around the impacting center so that it will divert the liquid radially outward to conserve the mass, resulting in the formation of the kink that has a sharp curvature. Subsequently, pushed by the ensuing liquid, the kink would eventually touch the substrate, initiating the spreading process.

At *t* = 0.5 ms, the droplet shape as a whole displays no distinct difference among the four. Nevertheless, for the outcome in the finer gird, a number of small holes appear, whose presence could be seen as the precursor of splashing. As time progress to *t* = 1.0 ms, the small holes show up in all the four columns, and several daughter droplets emerge in the coarser grid with a contact angle of 140°. At *t* = 1.3 ms, the protrusion around the edge grows bigger due to the inertia, a phenomenon that is more evident in the finer grid.

Thus far, the comparisons between outcomes on different meshes and between the experimental and the numerical, have exhibited reasonable agreement. As computation in three dimensions is prohibitive, the mesh of ∆*x* = 5 × 10^−5^ m is adopted in the following simulations. This resolution corresponds to 36 cells across droplet diameter in this section, and 40 cells across droplet diameter in Section 3.3, and 54 cells across the effective droplet diameter in Section 3.4 and Section 3.5.

**Figure 7:**
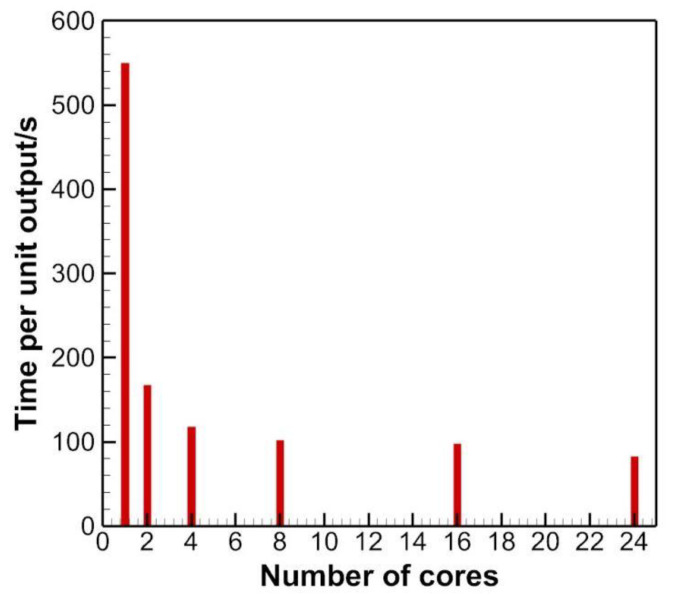
Efficiency of the parallel algorithm.

Figure 7 gives the efficiency of the parallel algorithm for the mesh of 131 × 101 × 131. The time per unit output means the time needed to output the outcome every 100∆*t,* where ∆*t* is the time step.

Figure 7 shows that when the code is run in a serial fashion, the time per unit output is roughly 550 s. As the number of cores doubles, the time rapidly reduces to about 170 s, plummeting by a factor of 3, which is quite satisfying. However, as the number continues going up, the reduction slows down, probably due to the overhead in synchronization when a parallel region is ended, and a serial region is to be en-counted.

### 3.3. Validation of the Model

**Figure 8:**
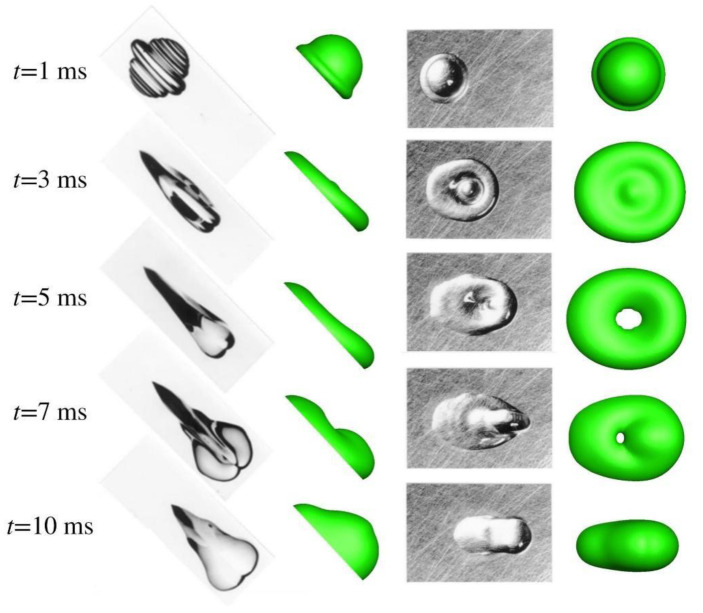
Oblique impact (45°) of water droplet. The left is the side view, and the right is the top view.

In Section 3.1, inertial force dominates in the time scale concerned, and the droplet impacts at a normal angle with the substrate. Herein, an oblique impact is considered, lasting to such a time scale that surface tension matters. This thus helps validate the model from a different perspective. The water droplet has a diameter of 2 mm, impacting at 1 m/s onto an incline making an angle of 45° with the substrate. The computational domain extends to 2.5 mm × 3 mm × 7 mm in the *x*, *y*, and *z* directions, respectively. The number of grid points reaches 51 × 61 × 151. Besides, it is worth noting that azimuthal symmetry existing no longer, the number refers to that in half of the computational domain. The contact angle is set to 90° and the numerical outcome is given in Figure 8. The experiment could be consulted in [25].

Figure 8 unveils a variety of phenomena of interest. At *t* = 1 ms, the droplet is rushing down, spurred by inertia and gravity. A rim forms around the periphery. The apparent contact angle, deemed as the contact angle perceived on a macro scale, differs in the advancing and the trailing edges, a phenomenon that is definitely caused by the pulling of gravity. In addition, the droplet profile in the top view verifies that azimuthal symmetry breaks. At *t* = 3 ms, the spreading continues. The difference in the apparent contact angle is amplified and could be perceived better. In the top view, the droplet is expanded upwards and downwards, presenting itself as a pancake. The bulge at the center of the pancake shows that spreading is still ongoing. Besides, the droplet takes on a non-spherical shape. At *t* = 5 ms, dragged by gravity and accommodated by surface tension, the rim thickness has been increased at the advancing edge while decreased at the trailing edge. In the top view, there exists a hole in the numerical outcome, which is absent in the experiment. Nevertheless, it is obvious from the experiment that in the middle is a thin film, which needs sufficient finer grids to resolve in simulations.

The dynamic evolution from 1 ms to 5 ms could be defined as the spreading phase, which features the conversion from kinetic energy to surface energy and to viscous dissipation. From then on, the retracting phase follows, characterized by the conversion from surface energy to kinetic energy. Since the droplet takes on a non-spherical shape, retraction progresses not at an even pace around the periphery. It goes on more swiftly in the direction of lateral expansion, and relatively slowly in the direction of downward motion, as gravity stretches the droplet, extending further accordingly. With retracting by surface tension and pulling by gravity carrying on, a finger pointing in the direction parallel to the surface of the substrate appears at *t* = 7 ms in the top view. After retraction completes in the direction perpendicular to the paper, it proceeds more prominently in the direction parallel to the substrate. This eventually leads to the droplet profile at *t* = 10 ms, without bouncing captured.

**Figure 9:**
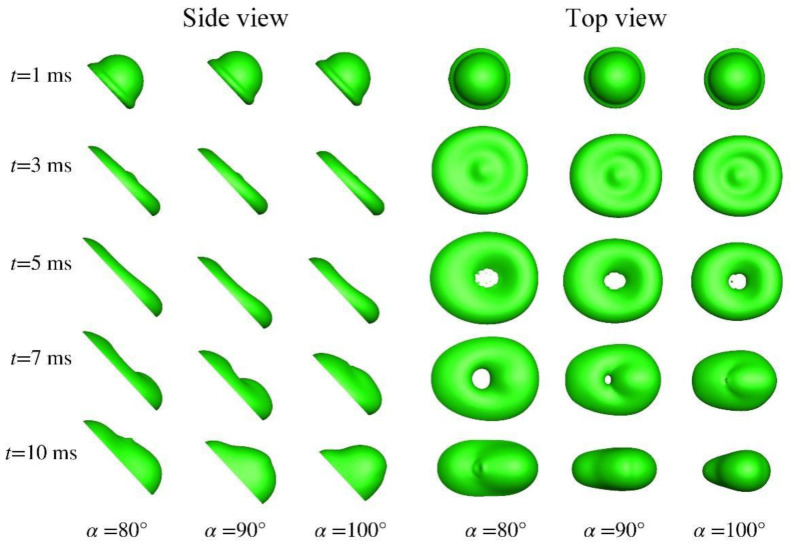
Contact angle effect on the oblique impact (45°) of water droplet.

Figure 9 gives the effect of contact angle, with three different contact angles being considered. At *t* = 3 ms, the drop at a hydrophilic substrate surface extends more readily than at a hydrophobic surface, which has been recognized by many researchers, no matter in normal or oblique impacts [26,27,28]. As the droplet extends farther when *α* = 80°, it takes more time for surface tension to initiate the retracting process. In opposite is the case where *α* = 100°, the evolution of which is basically the same with the others. However, due to its water repellent nature, the drop could extend no further than the case with *α* = 80°.

**Figure 10:**
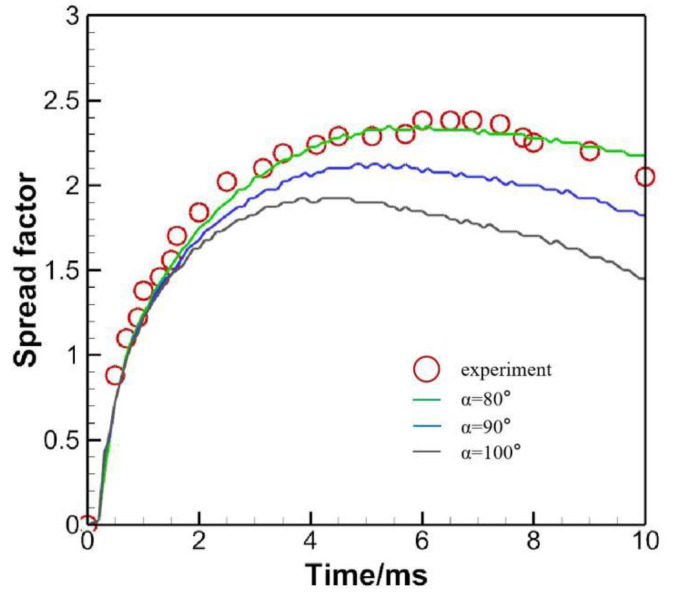
Spread factor evolution compared with the experiment.

To quantitatively measure the numerical model, the spread factor measured from the leading to trailing edge is extracted and compared with the experimental value, as shown in Figure 10. It is demonstrated that the numerical outcome agrees quite well with the experimental one when the static contact angle is set to 80°.

To sum up, the conservative level set model developed by [10] could be utilized to simulate droplet impingement at different impacting angles and at a large density and viscosity contrast. The method boasts of a second order interfacial evolution equation, called the conservative Allen-Cahn equation, whose counterpart is a fourth order Cahn-Hilliard equation. It thus embraces less severe time step requirements in explicit finite difference methods.

### 3.4. Oblate Droplet Impingement with a 90° Impacting Angle

**Figure 11:**
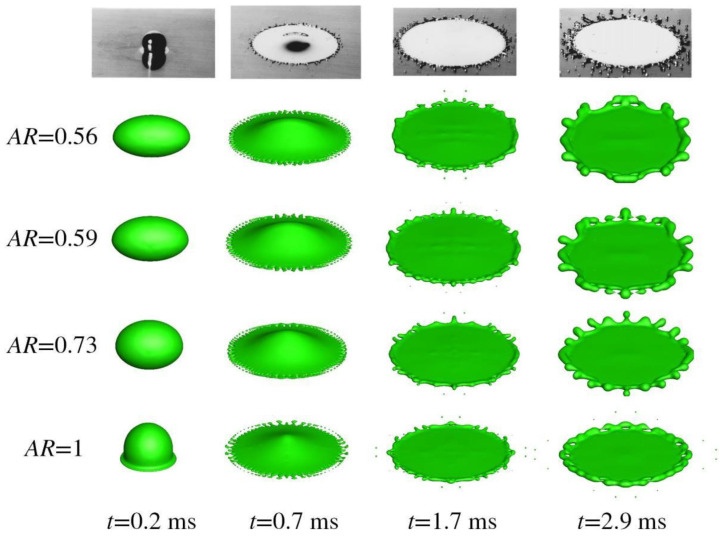
Oblate Tin droplet impingement with a 90° impacting angle.

This section deals with oblate droplet impact with splashing dynamics. Its geometric aspect ratio (AR), defined as the ratio of the maximal extension in the *y* axis to that in the *x* axis, may affect the number of fingers in case of splashing and droplet profile. Figure 11 presents numerical outcomes on oblate droplet impacts with different ARs.

The top row in the figure is the experimental outcome, which could be found in [29]. A total of four cases are displayed, with the AR ranging from 0.56 to 0.59 to 0.73 to 1 from top to bottom. It is to be noted that all of the four oblates have an identical coordinate for the origin initially, that the impacting velocity reaches 4 m/s, and that the contact angle is set to 140°. Besides, the number of grid points comes to be 171 × 101 × 171, with 12 cores mobilized to perform calculations in a parallel fashion.

**Figure 12:**
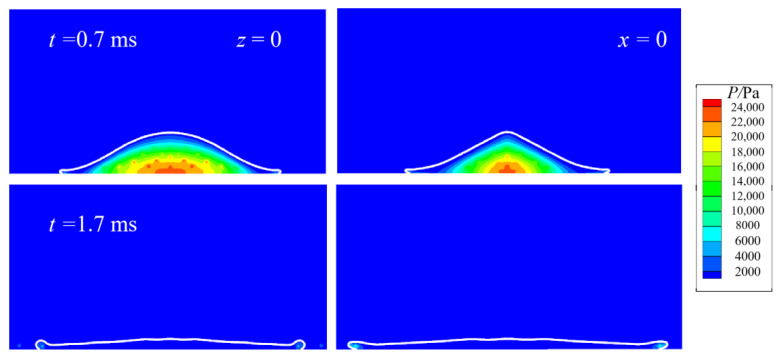
Pressure distribution in the cross-sectional area of *z* = 0 and *x* = 0 for *AR* = 0.56.

Inspection of the last row in the figure shows that reasonable agreement is achieved between the numerical and experimental. At *t* = 0.2 ms, a thin film develops around the periphery of the droplet, and its further radial movement initiates the spreading process. At *t* = 0.7 ms, the droplet is clearly moving outwards, but with significant ejecting of a number of daughter droplets around the edge. This type of splashing could be deemed as prompt splashing [30], which occurs in early stages of spreading and is contrary to the corona splashing that characterizes large angles the spreading front makes with a substrate. As spreading proceeds, the ejected daughter droplets would fly further, and the number of newly emerged fingers would be drastically reduced because of the enhanced role of surface tension in late stages of spreading, as shown at *t* = 1.7 ms. Subsequently, recoiling would take place, as demonstrated in the thickened edge at *t* = 2.9 ms. Figure 12 gives the pressure distribution in the cross-sectional area of *z* = 0 and *x* = 0 for *AR* = 0.56. The irregularities may come from the non-vanishing gradient of the level set away from the interface, or from the symmetry boundary condition set therein, which needs further study.

For a typical oblate droplet impact, as in Figure 11, the change of the AR alters mainly the distribution of fingers around the perimeter. Since in the major axis there is a sharp curvature, the Laplace pressure developed therein would hinder the initiation of surface instability, which may be seen as the precursor of splashing. While in the minor axis, the developed Laplace pressure is weakened compared with that for a spherical droplet so that splashing is liable to take place. Inspection of the last column in Figure 11 gives that the fingers concentrate on the longitudinal direction*,* while in the transverse direction the number is reduced heavily. Besides, as the AR is increased, finger distribution becomes uniform and the merging of fingers is mitigated, as each and every finger is of an equal size for *AR* = 0.73 at *t* = 2.9 ms.

Assuming the formation of fingers is due to a Rayleigh-Taylor instability, the number of fingers for spherical droplet impact could be estimated using the formula [31]

(13) $$N=\frac{We^{0.5}Re^{0.25}}{4\sqrt{3}}=\frac{K}{4\sqrt{3}}$$

where $We=\rho_{l}DV^{2}/\sigma_{s}$ measures the relative importance between inertia and surface tension $Re=\rho_{l}DV/\mu_{l}$ measures that between inertia and viscous force. Herein, *We*~430 and *Re*~39,206, indicating that *N*~40. This is quite close to the numerical prediction by directly counting the number of fingers around the circumference for all the cases at *t* = 0.7 ms.

**Figure 13:**
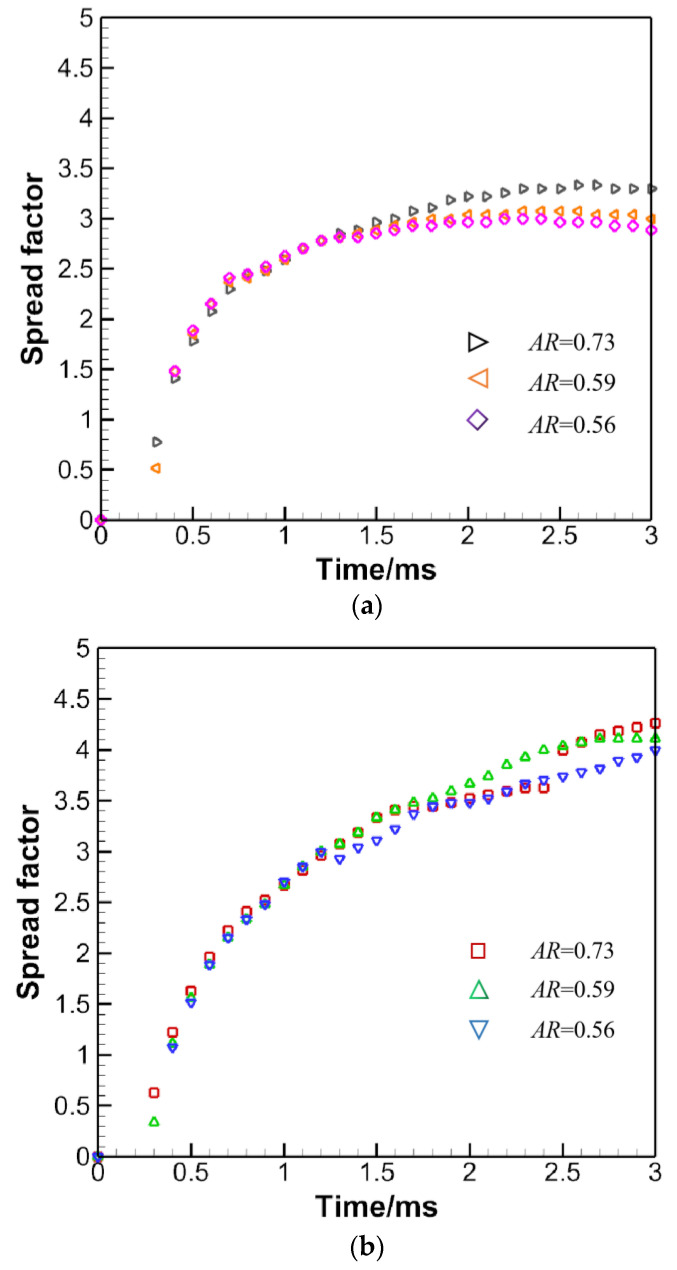
(**a**) Spread factor evolution for the oblate droplet impact in the transverse direction. (**b**) Spread factor evolution for the oblate droplet impact in the longitudinal direction.

The spread factor, defined as the ratio of the extension in the radial direction to the original droplet diameter, quantifies the extent to which the droplet expands. For spherical droplets, the spread factor is unequivocal, since the droplet spreads with azimuthal symmetry. Nevertheless, for non-spherical droplets, the spread factor may turn out to be varied if measured along different directions. Figure 13 yields the spread factor along the transverse (*x*-axis) and longitudinal (*z*-axis) directions.

**Figure 14:**
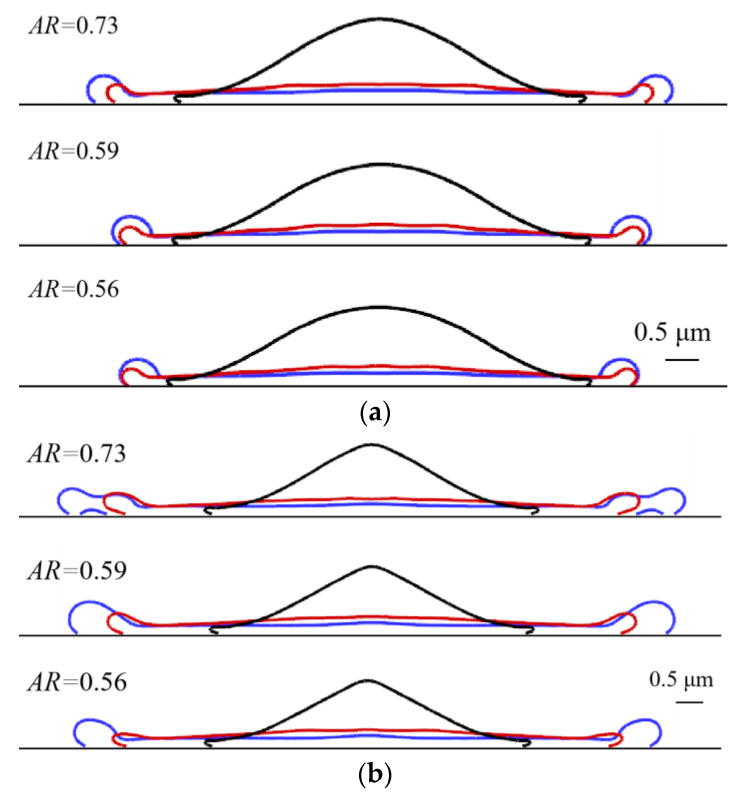
(**a**) Shapes of the cross-sectional area (*z* = 0) at various instants for different ARs. (**b**) Shapes of the cross-sectional area (*x* = 0) at various instants for different ARs. The black solid line denotes 0.7 ms, the red 1.7 ms and the blue 2.9 ms.

Upper is for the transverse direction, with the largest spread factor of around 3.4. The larger the AR, the bigger the spread factor. For the longitudinal direction, larger ARs lead to bigger spread factors as well. The maximal spread factor comes bigger in the longitudinal direction as expected, as demonstrated in Figure 14, which is drawn on the same scale, with the black solid line denoting 0.7 ms, the red 1.7 ms and the blue 2.9 ms. Besides, the sudden jump in the spread factor may be ascribed to the formation of fingers or the jetting of smaller droplets along the axis where the spread factor is tracked.

One interesting phenomenon is Figure 14b is the “air entrapment” for *AR* = 0.73 at 2.9 ms. This is actually not air entrapment, but the second touch of the spreading front with the substrate. If the simulation was run in two dimensions, the air entrapment would be predicted. While in three dimensions, this is just a through pore, which is formed by the retouch with the substrate. Normally, under this circumstance, the spreading front is on the brink of splashing. Nevertheless, it fails to jet satellite droplets and surface tension force could not pull it back to the main body so that the long liquid sheet retouches the substrate with a pore generated.

**Figure 15:**
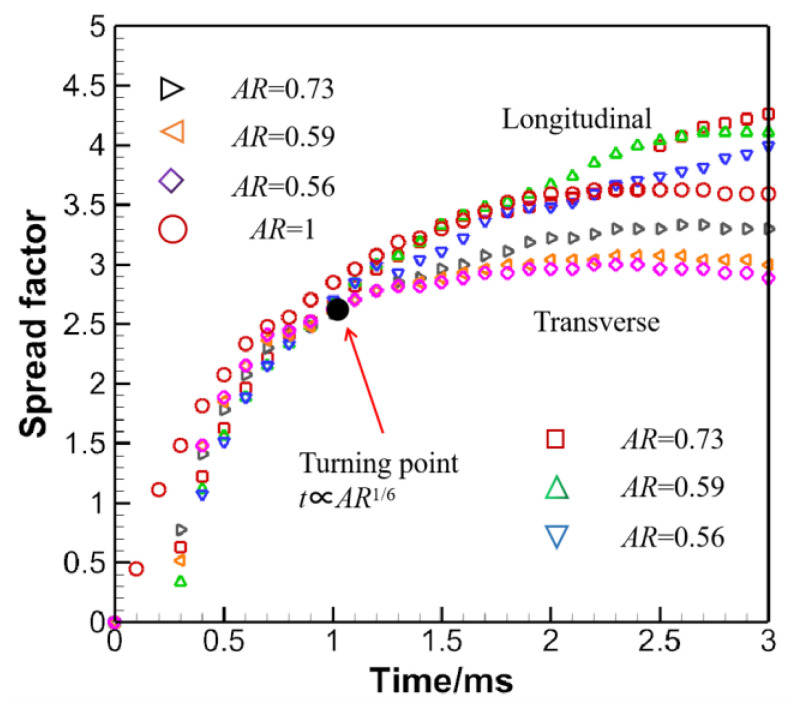
Comparison of spread factors of distinct ARs in different tracking directions.

Some additional lights could be shed if the spread factors of distinct ARs in different tracking directions are compared, as in Figure 15. It is found that before the turning point $t\propto AR^{1/6}$, the spread factors come bigger in the transverse direction. However, they turn smaller than those in the longitudinal direction after the turning point. Besides, the maximum spread factor *β_max_* for *AR* = 1 falls between those of the longitudinal and transverse directions. This trend is also in good agreement with the findings in [32].

**Figure 16:**
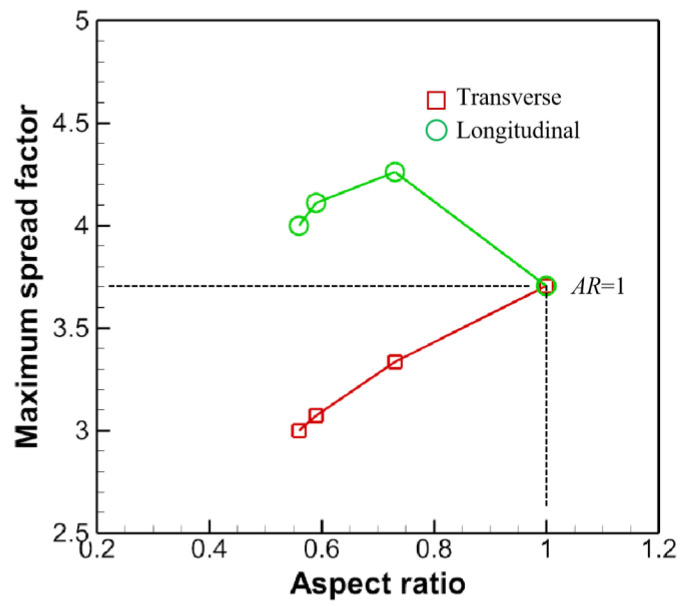
Maximum spread factors for different ARs for the oblate drop impact.

Figure 16 tells that for the transverse direction, *β_maxt_* increases almost linearly with the AR. While for the longitudinal direction, the curve behaves like a parabola, opening downwards. Accordingly, there probably exists a maximum value of *β_maxl_* therein, which is of interest for further studies. In this paper, the maximum could be sought around *AR* = 0.7. Besides, *β_maxl_/β_maxt_~AR^−^*^1/2^ [32], where *AR* is defined differently in [32]. The subscripts *maxl* and *maxt* stand for the maximum spread factors in the longitudinal and the transverse directions, respectively.

### 3.5. Prolate Droplet Impingement with an 90° Impacting Angle

**Figure 17:**
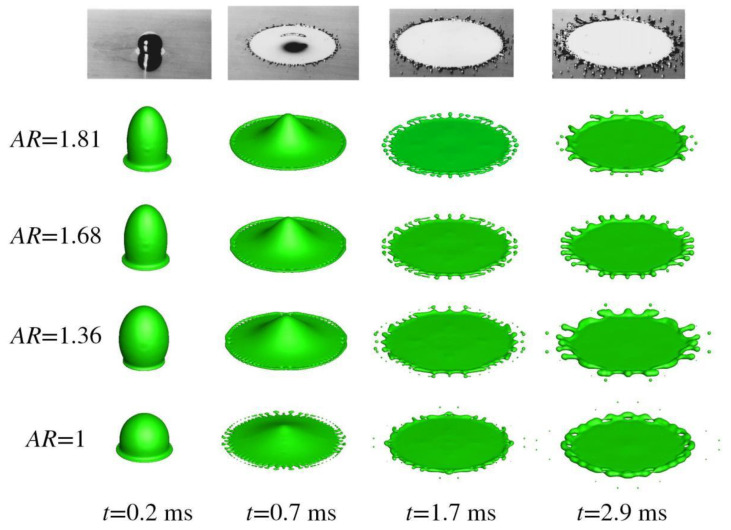
Prolate Tin droplet impingement with an 90° impacting angle.

Prolate is another non-spherical shape occurring in industrial applications. Prolate shape may help an air bubble rise in a water solution, since the drag coefficient could be dramatically decreased. This special shape could be realized by introducing electric or magnetic force along an interface, which will stretch the interface in the direction of the applied field [33]. Figure 17 presents the numerical outcome on prolate droplet impact. It is to be noted that the AR decreases from 1.81 to 1.68 to 1.36 to 1 from top to bottom, that the origin of the prolate is the same, and that the number of grid points is still 171 × 101 × 171. The other numerical configurations are consulted in Section 3.3.

**Figure 18:**
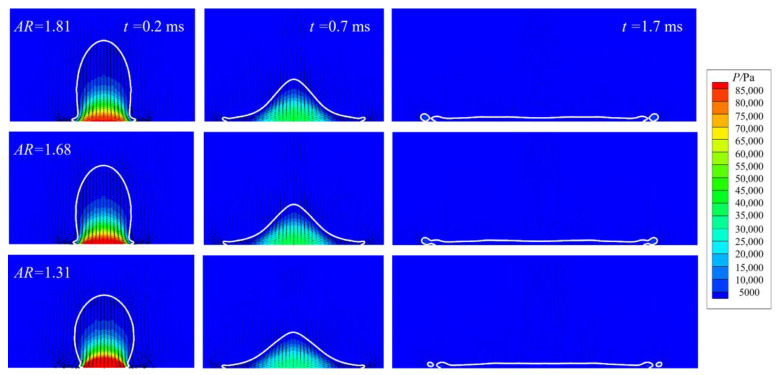
Pressure evolution and streamline distribution during early stages of spreading.

As azimuthal symmetry does exist in prolate droplet impact, the spreading process is the same regardless of the azimuthal angle. The major difference with increased AR is at *t* = 0.7 ms. For *AR* = 1.81, there are a number of small holes developed along the circumference, without significant jetting of daughter droplets. However, as the AR is decreased, the jetting is obvious, especially in the spherical case where the AR is unity. This could be explained as follows: when the AR is large, namely, when the droplet approaches the substrate with smaller bottom area like a pin, air trapping would be mitigated drastically, leading to a sluggish stagnation pressure, compared with a droplet impact with a smaller bottom curvature. Therefore, air cushioning is reduced as well, with the boundary layer developing earlier than for the normal spherical case so that more kinetic energy is dissipated, and splashing is less likely at an early stage. When splashing actually occurs at *t* = 1.7 ms, Equation (13) could still give a satisfying estimate of the number of fingers by comparing it with the numerical prediction. This kind of non-spherical impact shows that prompt splashing, occurring in early stages of spreading, could be controlled. Figure 18 gives the pressure and streamline distribution.

**Figure 19:**
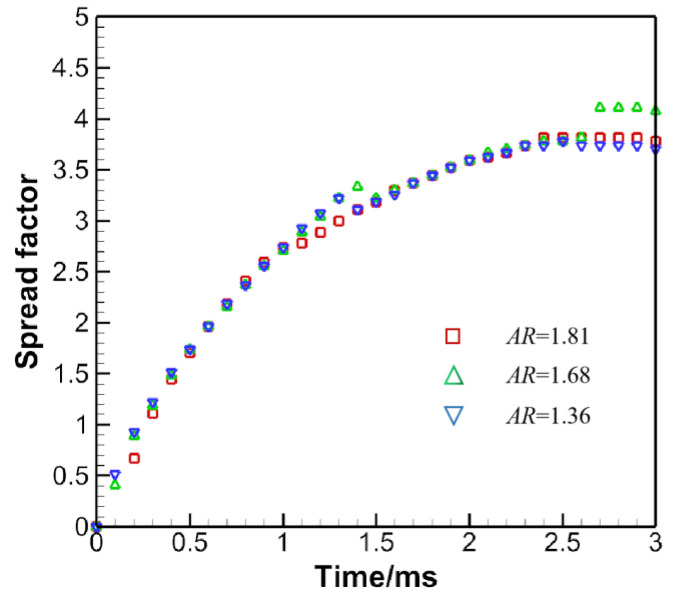
Spread factor evolution for the prolate droplet impact.

As the drop approaches the substrate, pressure would build up beneath the drop, resulting in a large pressure gradient along the substrate surface to drive contact line motion. Nevertheless, pressure diminishes rapidly, becoming uniform as shown at 0.17 ms. Figure 19 gives the spread factor evolution.

**Figure 20:**
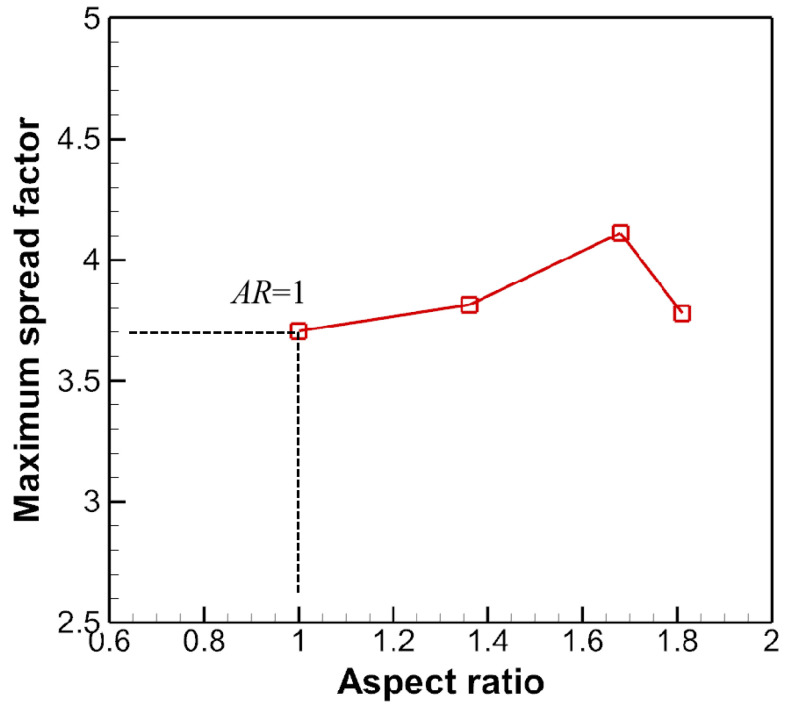
Maximum spread factors for different ARs for the prolate drop impact.

Figure 19 shows that in early stages of spreading, the spreading factor is nearly the same, indicating the dominant role of inertia over capillary force. This regime is often denoted as the kinematic regime, where material properties and surface wettabilities play negligible roles. Besides, as the AR is increased, the maximum spread factor is on the rise as well, although to a lesser degree. The sudden jump in the spread factor for *AR* = 1.68 may be ascribed to the formation of fingers or the jetting of smaller droplets along the axis where the spread factor is tracked. Graphing the maximum spread factor with respect to the AR in Figure 20 shows a slope larger than zero before *AR* < 1.6, which is consistent with the findings in [34].

The authors in [34] developed a correlation based on the energy analysis relating the maximum spread factor to the aspect ratio, taking the form

(14) $$\beta_{max}=AR^{1/6}\sqrt{\frac{We+BoAR^{2/3}+12S_{A}}{3\left(1-\cos\theta_{s}\right)AR^{1/3}+4We/\sqrt{Re}}}$$

where $Bo=∆\rho gD^{2}/\sigma_{s}\sim O\left(1\right)$, measuring the relative importance of gravity against surface tension, and $4We/\sqrt{Re}\sim O\left(10\right)$ in this paper. $\theta_{s}$ is set to be 140° herein. $S_{A}$ is given by [35]

(15) $$S_{A}=\{\begin{array}{ccc} \frac{1}{2AR^{2/3}}\left(1+\frac{AR^{2}\mathrm{arctanh}\;e}{e}\right) & e=\sqrt{1-AR^{2}}, & AR<1,\\ 1, & e=0, & AR=1,\\ \frac{1}{2AR^{2/3}}\left(1+\frac{AR\arcsin\;e}{e}\right) & e=\sqrt{1-1/AR^{2}}, & AR>1, \end{array}$$

**Figure 21:**
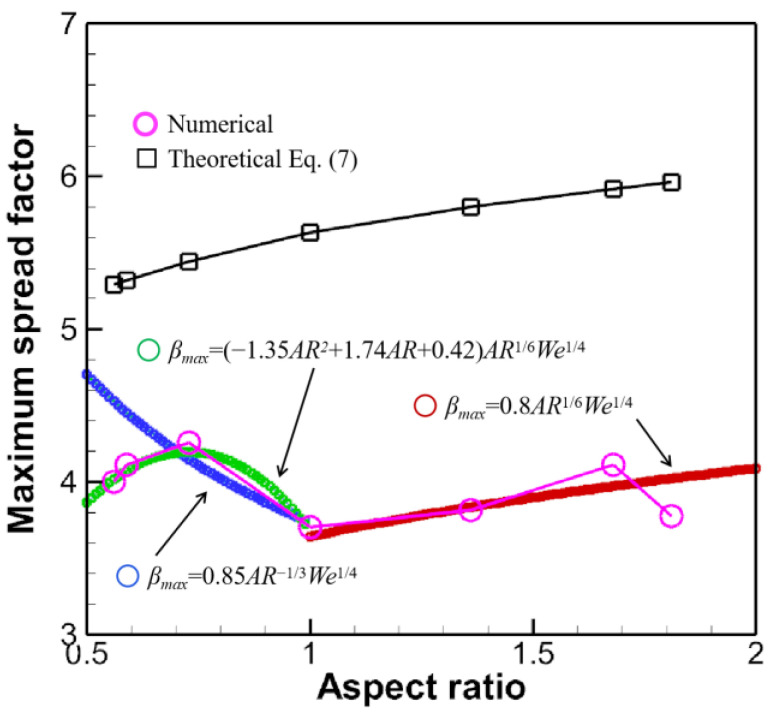
Maximum spread factors for different ARs. The oblate droplet impact corresponds to those ARs smaller than unity, while the prolate droplet impact to those larger than unity.

The calculated $S_{A}$ is listed in Table 2. The computed $\beta_{max}$ is graphed and compared with the numerical ones in Figure 21, which indicates a clear overshoot of the theoretical model.

It is to be noted that in Figure 21 the maximum spread factors for the oblate drop impact lie in the longitudinal direction. Another commonly used formula for the maximum spread factor $\beta_{max}$ is concerned with the *We* number, scaling as $\beta_{max}\sim We^{1/4}$ [36,37], if the droplet is said to be in the capillary regime if $We/Re^{4/5}<1$. In the capillary regime, viscous dissipation is negligible, while the kinetic energy is converted almost entirely into surface energy. Notice that this scaling is for spherical droplets. To take into account morphology effect, $\beta_{max}$ is interpolated to obey the power law

(16) $$\beta_{max}=\{\begin{array}{cc} \left(-1.35AR^{2}+1.74AR+0.42\right)AR^{1/6}We^{1/4}, & AR<1,\\ 0.8AR^{1/6}We^{1/4}, & AR\geq 1, \end{array}$$

which shows reasonable agreement with the numerical prediction denoted in the purple circle in Figure 21. The authors in [32] also put forward an empirical formula to predict the maximum spread factors for only oblate drop impact, scaling as ~*CxAR*^−1/3^*We*^1/4^, which agrees well in the range of *AR* < 0.7 but fails elsewhere. *Cx* is a fitting parameter on the order of unity and is adjusted to be 0.85 in Figure 21.

## 4. Concluding Remarks

A conservative level set model, second order in nature, prevails over the Cahn-Hilliard based phase field model, fourth order per se, in terms of the explicit finite difference solutions. This paper utilizes such a conservative level set model to predict non-spherical drop impact and splashing dynamics. The advection term in the conservative level set model was tackled using the traditional central difference scheme. The major findings are as follows. For oblate drop impact, the number of satellite droplets in terms of their first appearance are counted to be in line with an empirical formula. Besides, the future evolution of the fingers is more likely to be inhibited on the edges of sharper curvatures. For prolate drop impact, the impacting and spreading processes are still azimuthally symmetric, but the fingers turn out to be larger and penetrate farther into the surrounding gas as the aspect ratio increases. Besides, the prompt slashing occurring in the early stages of spreading could be inhibited by adjusting the shape of the impacting droplet.

## Figures and Tables

**Table 1:** Thermophysical quantities and model parameters employed in this paper.

Parameters	Tin	Water	Air
Density $\;\left(\mathrm{kg}/m^{3}\right)$	6970	1000	1.18
Viscosity (mPa·s)	1.92	1	0.0185
Surface energy (N/m)	0.7	0.072	
Re-initialization strength *ω* (m/s)	*V*	*V*	
Interface thickness ϵ (m)	$5\times 10^{-5}$	$5\times 10^{-5}$	
Spatial step ∆x=∆y=∆z (m)	$5\times 10^{-5}$	$5\times 10^{-5}$	

**Table 2:** Calculated *S_A_* for various ARs.

AR	$S_{A}\;(\mathrm{Oblate})$	AR	$S_{A}\;(\mathrm{Prolate})$
0.56	1.02	1.36	0.94
0.59	1.01	1.68	0.91
0.73	0.97	1.81	0.91

## Abbreviations

**List of Symbols**	
u	Velocity (m/s)
*t*	Time (s)
*p*	Pressure (Pa)
***ρ***	Density (${\mathrm{kg}/m}^{3}$)
**σ**	Newtonian stress tensor (Pa)
**g**	Gravitational acceleration (${m/s}^{2}$)
*σ* *_s_*	Surface tension coefficient (N/m)
μ	Viscosity (Pa•s)
ϵ	Characteristic interfacial thickness (m)
***ω***	Re-initialization strength (m/s)
$F_{lsm}$	Surface tension force (N/m^3^)
n	Unit normal
Δ*x*	Spatial step (m)
Δ*y*	Spatial step (m)
Δ*z*	Spatial step (m)
*i*	Coordinate index
*j*	Coordinate index
*k*	Coordinate index
*λ*	Relaxation factor
*p^i^*	The *i*th iteration value of *p*
*L* *X*	Computational length in the *x* direction
*L* *Y*	Computational length in the *y* direction
*L* *Z*	Computational length in the *z* direction
*α*	Contact angle (°)
*β_max_*	Maximum spread factor
*β_maxl_*	Maximum spread factor in the longitudinal direction
*β_maxt_*	Maximum spread factor in the transverse direction
*Re*	Reynolds number
*We*	Weber number
*Bo*	Bond number
*AR*	Aspect ratio
**Subscript**	
*t*	Partial derivative with respect to time
*x/y/z*	Partial derivative with respect to *x/y/z*
**Superscript**	
*n*	Previous time level
*n* + 1	Current time level
*i*	The *i*th iteration

## References

[1] Burzynski D.A., Roisman I.V., Bansmer S.E. (2020). On the splashing of high-speed drops impacting a dry surface. J. Fluid Mech..

[2] de Goede T., de Bruin K., Shahidzadeh N., Bonn D. (2021). Droplet splashing on rough surfaces. Phys. Rev. Fluids.

[3] Zhang Y., Matthews S., Wu D., Zou Y. (2022). Interactions between successive high-velocity impact droplets during plasma spraying. Surf. Coat. Technol..

[4] Zhang H., Zhang X., Yi X., Du Y., He F., Niu F., Hao P. (2022). How surface roughness promotes or suppresses drop splash. Phys. Fluids.

[5] Anjos G. (2021). Moving Mesh Methods for Two-Phase Flow Systems: Assessment, Comparison and Analysis. Comput. Fluids.

[6] Liu L.T., Chen X.B., Zhang W.Q., Zhang A.-M. (2020). Study on the transient characteristics of pulsation bubble near a free surface based on finite volume method and front tracking method. Phys. Fluids.

[7] Boniou V., Schmitt T., Vié A. (2022). Comparison of interface capturing methods for the simulation of two-phase flow in a unified low-Mach framework. Int. J. Multiph. Flow.

[8] Osher S., Sethian J.A. (1988). Fronts propagating with curvature-dependent speed: Algorithms based on Hamilton-Jacobi formulations. J. Comput. Phys..

[9] Olsson E., Kreiss G. (2005). A conservative level set method for two phase flow. J. Comput. Phys..

[10] Chiu P.-H., Lin Y.-T. (2011). A conservative phase field method for solving incompressible two-phase flows. J. Comput. Phys..

[11] Jain S.S., Mani A., Moin P. (2020). A conservative diffuse-interface method for compressible two-phase flows. J. Comput. Phys..

[12] Howard A.A., Tartakovsky A.M. (2021). A conservative level set method for N-phase flows with a free-energy-based surface tension model. J. Comput. Phys..

[13] Mirjalili S., Jain S.S., Mani A. (2022). A computational model for interfacial heat and mass transfer in two-phase flows using a phase field method. Int. J. Heat Mass Transf..

[14] Huang J.-J., Zhang L. (2022). Simplified method for wetting on curved boundaries in conservative phase-field lattice-Boltzmann simulation of two-phase flows with large density ratios. Phys. Fluids.

[15] Singh S., Saha A. (2022). Numerical study of heat transfer during oblique impact of a cold drop on a heated liquid film. J. Therm. Sci. Eng. Appl..

[16] Qu J., Yang Y., Yang S., Hu D., Qiu H. (2019). Droplet impingement on nano-textured superhydrophobic surface: Experimental and numerical study. Appl. Surf. Sci..

[17] Yun S., Kim I. (2019). Spreading Dynamics and the Residence Time of Ellipsoidal Drops on a Solid Surface. Langmuir.

[18] Yun S. (2021). Ellipsoidal drop impact on a single-ridge superhydrophobic surface. Int. J. Mech. Sci..

[19] Mirjalili S., Ivey C.B., Mani A. (2019). A conservative diffuse interface method for two-phase flows with provable boundedness properties. J. Comput. Phys..

[20] Ding H., Spelt P.D.M. (2007). Wetting condition in diffuse interface simulations of contact line motion. Phys. Rev. E.

[21] Lee H.G., Kim J. (2011). Accurate contact angle boundary conditions for the Cahn–Hilliard equations. Comput. Fluids.

[22] Emdadi M., Pournaderi P. (2019). Numerical simulation of conducting droplet impact on a surface under an electric field. Acta Mech..

[23] Göhl J., Mark A., Sasic S., Edelvik F. (2018). An immersed boundary based dynamic contact angle framework for handling complex surfaces of mixed wettabilities. Int. J. Multiph. Flow.

[24] Tsai P., Pacheco S., Pirat C., Lefferts L., Lohse D. (2009). Drop Impact upon Micro- and Nanostructured Superhydrophobic Surfaces. Langmuir.

[25] Bussmann M., Mostaghimi J., Chandra S. (1999). On a three-dimensional volume tracking model of droplet impact. Phys. Fluids.

[26] Lyu J., Gao L., Zhang Y., Bai M., Li Y., Gao D., Hu C. (2021). Dynamic spreading characteristics of droplet on the hydrophobic surface with microstructures. Colloids Surf. A Physicochem. Eng. Asp..

[27] Shusheng Z., Hao L., Li-Zhi Z., Saffa R., Zafer U., Huaguan Z. (2020). A lattice Boltzmann simulation of oblique impact of a single rain droplet on super-hydrophobic surface with randomly distributed rough structures. Int. J. Low-Carbon Technol..

[28] Wang Y.-B., Gao S.-R., Yang Y.-R., Wang X.-D., Chen M. (2020). Universal Model for the Maximum Spreading Factor of Impacting Nanodroplets: From Hydrophilic to Hydrophobic Surfaces. Langmuir.

[29] Aziz S.D., Chandra S. (2000). Impact, recoil and splashing of molten metal droplets. Int. J. Heat Mass Transf..

[30] Yu F., Lin S., Yang J., Fan Y., Wang D., Chen L., Deng X. (2020). Prompting Splash Impact on Superamphiphobic Surfaces by Imposing a Viscous Part. Adv. Sci..

[31] Bussmann M., Chandra S., Mostaghimi J. (2000). Modeling the splash of a droplet impacting a solid surface. Phys. Fluids.

[32] Yun S., Lim G. (2014). Ellipsoidal drop impact on a solid surface for rebound suppression. J. Fluid Mech..

[33] Yang Q., Li B.Q., Shao J., Ding Y. (2014). A phase field numerical study of 3D bubble rising in viscous fluids under an electric field. Int. J. Heat Mass Transf..

[34] Zhang X., Ji B., Liu X., Ding S., Wu X., Min J. (2021). Maximum spreading and energy analysis of ellipsoidal impact droplets. Phys. Fluids.

[35] Zwillinger D. (2018). CRC Standard Mathematical Tables and Formulas.

[36] Clanet C., Béguin C., Richard D., Quéré D. (2004). Maximal deformation of an impacting drop. J. Fluid Mech..

[37] Laan N., de Bruin K.G., Bartolo D., Josserand C., Bonn D. (2014). Maximum Diameter of Impacting Liquid Droplets. Phys. Rev. Appl..

